# Cancer risk in patients with hepatitis C virus infection: a population‐based study in Sweden

**DOI:** 10.1002/cam4.988

**Published:** 2017-04-04

**Authors:** Xiangdong Liu, Yanqing Chen, Youxin Wang, Xiaohua Dong, Junming Wang, Jianhua Tang, Kristina Sundquist, Jan Sundquist, Jianguang Ji

**Affiliations:** ^1^ Department of Poliomyelitis National Institute for Viral Disease Control and Prevention Chinese Center for Disease Control and Prevention Beijing China; ^2^ Center for Primary Health Care Research Lund University Malmö Sweden; ^3^ Beijing Huilongguan Hospital Beijng China; ^4^ School of Public Health Capital Medical University Beijing China; ^5^ Department of Pharmacology School of Pharmacy Hebei North University Zhangjiakou China; ^6^ College of Lab Medicine Hebei North University Zhangjiakou China; ^7^ Department of Clinical Pharmacology First Affiliated Hospital of Hebei North University Zhangjiakou China; ^8^ Stanford Prevention Research Center Stanford University School of Medicine California

**Keywords:** Cancer, hepatitis C virus, risk

## Abstract

Increased risks of certain cancers have been observed in patients with hepatitis C virus (HCV) infection. However, data on other cancer sites/types are lacking. We analyzed systematically the risk of developing 35 common cancers in patients with HCV infection using a nationwide Swedish database. Patients with HCV infection were identified from the Swedish Hospital Inpatient and Outpatient Register and Primary Health Care Database, and followed until the diagnosis of cancer. Standardized incidence ratios (SIRs) were calculated for subsequent 35 common cancer sites/types between 1990 and 2010 in patients with HCV infection in Sweden. Increased risks were recorded for six cancers. The highest SIR was seen for liver cancer (36.67; 95% CI: 33.20–40.40). The decreased risk was for prostate cancer (0.73; 95% CI: 0.59–0.90) and melanoma (0.50; 95% CI: 0.30–0.79). A significant sex‐difference for cancer was observed only for liver cancer (40.72; 95% CI: 36.36–45.45 for men and 27.21; 95% CI: 21.90–33.41 for women). Also, increased SIRs were noted only for liver cancer during the entire period of follow‐up. HCV infection was associated with an increased incidence of liver cancer and additionally five other types of cancer. Active surveillance of other cancers may be needed in order to be diagnosed at an earlier stage.

## Introduction

Hepatitis C virus (HCV) is a RNA virus of the Flaviviridae family, and its infection is considered as a major cause of chronic liver diseases, with potential for neoplastic degeneration. The estimated prevalence of HCV infection worldwide is 3% (> 170 million people) 1. In Europe, chronic hepatitis C affects about 9 million people and HCV infection causes 86,000 deaths each year 2. The prevalence of HCV infection is 0.59% in the general Swedish population 3. In recent years, the incidence of HCV has decreased gradually, mainly the HCV cases due to sexual contact 4. Various HCV proteins include core, envelope, and nonstructural proteins, which have oncogenic properties by inducing oxidative stress, disturbing cellular regulatory pathways associated with proliferation and apoptosis, and suppressing host immune response 5.

A number of epidemiological and experimental studies indicated that about 15–20% of human cancers are associated with chronic infection in some way 6. An increasing number of epidemiological studies have indicated that risks of several common human malignancies, including cancers of the liver, pancreas, and non‐Hodgkin lymphoma (NHL) is positively associated with HCV infection 5, 7, 8. HCV infection caused around 10% of cirrhosis and 12–17% of HCC in Korea 9, 10, 11. However, the association between HCV and other cancers is still lacking. In countries with high rates of HCV infection, studies examining associations with other cancer sites are usually unreliable because of the poor quality of cancer register. Conversely, the countries with high‐quality cancer registration have usually low prevalence of HCV infection. To explore this knowledge gap, we plan to systematically study the risk of 35 common cancers among patients with HCV infection by using the nationwide Swedish database, which might successfully avoid the difficulties described above. The novelty of this study is the systematic assessment of the association between HCV infection and risk of 35 common cancer sites/types using a nationwide cohort including most patients with HCV infection treated in hospital and primary health care in Sweden.

## Material and Methods

The research database used in this study is nationwide and covers the entire population of Sweden over a defined period of time (a total of 14.4 million living and deceased individuals until 2010), and is maintained at the Center for Primary Health Care Research, Lund University, Malmö, Sweden. It was created using data from several national Swedish registers provided by Statistics Sweden. The data on HCV infection were achieved from the Swedish Hospital Discharge Register, the Swedish Outpatient Register and the primary health care in Stockholm and Region Skåne. Dates of hospitalization and diagnoses are recorded in the Swedish Hospital Discharge Register regionally since 1964 and nationwide since 1986. The National Board of Health and Welfare created the Swedish Outpatient Register in 2001 and the coverage of public hospitals was close to 100%. The Primary Health Care Registry in Stockholm covers 75 primary health care centers in Stockholm and middle of Sweden from January 1, 2001 to June 30, 2007 and contains information on medical diagnoses in these centers. The data on all medical diagnoses in the primary health care centers in Region Skåne are included in the Primary Health Care Registry in Region Skåne from 1973 to 2010. We used the International Classification of Diseases codes to identify the patients with HCV infection. The patients with both HBV and HCV infections were excludes in this study because HBV is a known risk factor for many cancers 12. The diagnosis of HCV in Sweden was started in 1990 when the first generation of diagnostic tests for anti‐HCV was available. The second‐generation anti‐HCV assays were introduced in 1991, and later with HCV‐RNA analysis by real‐time PCR 13. We achieved the individual information on socioeconomic status by the linkages to national census data, identified the date of death by the linkage to the Cause of Death Register and obtained the date of emigration by the linkage to the Emigration Registry. We made all linkages via an individual national identification number. This number was assigned to each person for their lifetime in Sweden and was replaced by a serial number providing anonymity.

The data on cancer were obtained from the nationwide Swedish Cancer Registry. This study excluded the patients with HCV infection who had a previous cancer diagnosis. We calculated person‐years of follow‐up from the date of discharge of HCV infection (as the first main diagnosis) to cancer diagnosis, death, emigration, or closing date (December 31, 2010), whichever occurred first. We used the indirect standardization method to calculate standardized incidence ratio (SIR), which is the ratio of observed (O) to expected (E) numbers of cases:

$$\mathrm{SIR}=\frac{\sum_{j=1}^{J}o_{j}}{\sum_{j=1}^{J}n_{j}\lambda_{j}^{*}}=\frac{O}{E^{*}},$$

where *O*=∑*o*_*j*_ represents the total number of observed cases of cancer after HCV infection from the study group; *E* ^*^ (expected number of cancer cases) is calculated by applying stratum‐specific standard incidence rates (*λ* ^*^_j_) achieved from the reference group (all patients without HCV infection) to person‐years at risk for the specific stratum (*n*_*j*_) in the study group; *O*_*j*_ denotes the observed number of cases of the *j*th stratum contributed by cohort subjects; and *J* denotes the strata defined by the cross‐classification of several adjustment variables, including age (5‐year groups), sex, time period (5‐year groups), socioeconomic status (6 groups: farmers, unskilled/skilled workers, white‐collar workers, professionals, self‐employed, and all others), and geographic region of residence (3 groups: large cities [Stockholm, Gothenburg, and Malmö], Southern Sweden, and Northern Sweden). The 95% confidence intervals (CIs) were calculated and rounded to two decimal places for SIR, assuming a Poisson distribution. In this study, the result is statistically significant if the 95% CI does not include 1.00.

SAS software (version 9.3; SAS Institute, Cary, N.C.) was used to perform all statistical analyses.

The regional ethical review board at Lund approved this study.

## Results

Totally, 29,271 individuals (19,002 men and 10,269 women) were diagnosed with HCV infection in Sweden between 1990 and 2010 (Table 1). Most patients (23,976) among this population were born in Sweden. The median age at diagnosis of HCV infection was 44 years.

**Table 1:** Basic characteristics of patients with HCV infection

Characteristics	*N* (%)
Age at HCV diagnosis, years	
Median	44
Quarter range	35–52
Gender	
Male	19,002 (64.9)
Female	10,269 (35.1)
Sum	29.271
Year of HCV diagnosis	
Median	2003
Quarter range	2001–2005
Birth country	
Sweden	23.976
Finland	1.183
Iran	355
Poland/Danzig	292

In Table 2, we show the risk of cancer after HCV infection, and only those with at least five cases of cancer after HCV infection were listed. The overall SIR after HCV infection was 1.77 (95% CI: 1.67–1.87). Among 35 cancer sites/types, 6 showed increased SIRs after diagnosis of HCV infection, including upper aerodigestive tract (2.62; 95% CI: 1.98–3.40), esophagus (2.07; 95% CI: 1.15–3.42), liver (36.67; 95% CI: 33.20–40.40), lung (2.07; 95% CI: 1.71–2.49), squamous cell carcinoma (SCC) (2.06; 95% CI: 1.49–2.77), and non‐Hodgkin's lymphoma (2.18; 95% CI: 1.62–2.87). Decreased SIRs were noted for only two cancers: prostate cancer (0.73; 95% CI: 0.59–0.90) and melanoma (0.50; 95% CI: 0.30–0.79). The overall risk of cancer after HCV infection was 1.94 (95% CI: 1.80–2.07) for men and 1.50 (95% CI: 1.35–1.66) for women. A significant sex‐difference for cancer was observed only for liver cancer (40.72; 95% CI: 36.36–45.45 for men and 27.21; 95% CI: 21.90–33.41 for women).

**Table 2:** SIRs in cancer patients after HCV infection by gender

Cancer site	Male			Female			All		
	O	SIR	95% CI	O	SIR	95% CI	O	SIR	95% CI
Upper aerodigestive tract	48	**2.68**	1.98–3.56	9	**2.34**	1.06–4.47	57	**2.62**	1.98–3.40
Esophagus	12	1.91	0.98–3.34	3	3.10	0.58–9.17	15	**2.07**	1.15–3.42
Stomach	7	0.78	0.31–1.62	5	1.60	0.51–3.77	12	0.99	0.51–1.74
Small intestine	4	1.65	0.43–4.27	2	2.11	0.20–7.75	6	1.78	0.64–3.90
Colon	26	1.05	0.69–1.54	11	0.82	0.41–1.47	37	0.97	0.68–1.34
Rectum	17	0.94	0.55–1.51	8	1.18	0.50–2.34	25	1.01	0.65–1.49
Anus	5	**4.25**	1.34–9.99	1	0.89	0.00–5.11	6	2.61	0.94–5.72
Liver	318	**40.72**	36.36–45.45	91	**27.21**	21.90–33.41	409	**36.67**	33.20–40.40
Pancreas	14	1.68	0.92–2.83	5	1.27	0.40–2.98	19	1.55	0.93–2.42
Lung	70	**1.86**	1.45–2.35	44	**2.52**	1.83–3.39	114	**2.07**	1.71–2.49
Breast	0			78	0.81	0.64–1.02	78	0.81	0.64–1.01
Cervix	0			13	1.47	0.78–2.53	13	1.47	0.78–2.53
Endometrium	0			9	0.69	0.31–1.32	9	0.69	0.31–1.32
Ovary	0			11	1.07	0.53–1.91	11	1.07	0.53–1.91
Prostate	93	**0.73**	0.59–0.90	0			93	**0.73**	0.59–.90
Testis	8	0.87	0.37–1.71	0			8	0.87	0.37–1.71
Kidney	18	1.38	0.82–2.19	6	1.54	0.56–3.38	24	1.42	0.91–2.12
Urinary bladder	28	1.14	0.76–1.65	7	1.55	0.61–3.21	35	1.20	0.84–1.67
Melanoma	12	**0.52**	0.27–0.91	7	**0.47**	0.19–0.98	19	**0.50**	0.30–0.79
Skin, squamous cell	26	**1.81**	1.18–2.66	17	**2.59**	1.51–4.16	43	**2.06**	1.49–2.77
Nervous system	20	1.32	0.80–2.04	10	0.98	0.47–1.80	30	1.18	0.80–1.69
Thyroid gland	2	0.78	0.07–2.86	4	0.94	0.24–2.42	6	0.88	0.32–1.92
Endocrine glands	4	0.71	0.18–1.84	9	1.54	0.70–2.93	13	1.13	0.60–1.94
Connective tissue	6	1.90	0.69–4.17	1	0.70	0.00–4.03	7	1.53	0.61–3.17
Non‐Hodgkin's lymphoma	38	**2.24**	1.59–3.08	13	**2.02**	1.07–3.46	51	**2.18**	1.62–2.87
Myeloma	8	1.50	0.64–2.98	5	2.27	0.72–5.33	13	1.73	0.92–2.96
Leukemia	13	0.97	0.51–1.66	6	1.00	0.36–2.20	19	0.98	0.59–1.53
All cancer	805	**1.94**	1.80–2.07	381	**1.50**	1.35–1.66	1186	**1.77**	1.67–1.87

Bold type indicates that the 95% CI does not include 1.00.

Pyrs, person‐years; O, observed; SIR, standardized incidence ratio; CI, confidence interval.

Person‐years is 177213.

In order to explore the impact of duration of follow‐up on cancer risk, we stratified the analyses by four follow‐up intervals: <1 year, 1–4 years, 5–9 years, and ≥10 years (Table 3). The increased overall cancer risk was found for each of the four follow‐up intervals (3.33, 95% CI: 2.93–3.77 for <1 year; 1.67, 95% CI: 1.53–1.82 for 1–4 years; 1.46, 95% CI: 1.32–1.62 for 5–9 years; and 1.46, 95% CI: 1.09–1.92 for ≥10 years). Only liver cancer showed increased SIRs during the whole follow‐up period (87.66, 95% CI: 72.30–105.33 for <1 year; 33.20, 95% CI: 28.41–38.58 for 1–4 years; 26.39, 95% CI: 21.65–31.87 for 5–9 years; and 26.16, 95% CI: 14.91–42.59 for ≥10 years).

**Table 3:** SIRs in cancer patients after HCV infection by follow‐up

Cancer site	Follow‐up (years)											
	<1			1–4			5–9			10+		
	O	SIR	95% CI	O	SIR	95% CI	O	SIR	95% CI	O	SIR	95% CI
Upper aerodigestive tract	5	2.04	0.64–4.79	22	2.18	1.36–3.30	29	3.63	2.43–5.22	1	0.84	0.00–4.81
Esophagus	2	2.51	0.24–9.22	3	0.90	0.17–2.67	9	3.31	1.50–6.31	1	2.40	0.00–13.76
Stomach	1	0.70	0.00–3.98	6	1.07	0.38–2.34	4	0.91	0.24–2.36	1	1.55	0.00–8.91
Small intestine	1	2.64	0.00–15.11	5	3.18	1.00–7.48	0			0		
Colon	5	1.15	0.36–2.69	15	0.85	0.47–1.40	14	0.99	0.54–1.67	3	1.49	0.28–4.40
Rectum	1	0.36	0.00–2.08	17	1.48	0.86–2.38	7	0.76	0.30–1.57	0		
Anus	2	7.83	0.74–28.78	3	2.81	0.53–8.32	0			1	7.94	0.00–45.51
Liver	114	87.66	72.30–105.33	171	33.20	28.41–38.58	108	26.39	21.65–31.87	16	26.16	14.91–42.59
Pancreas	9	6.50	2.95–12.40	3	0.53	0.10–1.58	7	1.53	0.60–3.16	0		
Lung	24	3.97	2.54–5.92	43	1.71	1.24–2.31	43	2.07	1.50–2.79	4	1.27	0.33–3.28
Breast	12	1.10	0.57–1.93	33	0.73	0.50–1.03	29	0.81	0.54–1.17	4	0.79	0.21–2.04
Cervix	1	0.88	0.00–5.02	5	1.16	0.36–2.72	4	1.35	0.35–3.49	3	7.67	1.45–22.70
Endometrium	1	0.70	0.00–4.00	5	0.84	0.27–1.99	3	0.61	0.11–1.80	0		
Ovary	1	0.83	0.00–4.76	9	1.88	0.85–3.57	1	0.26	0.00–1.52	0		
Prostate	12	0.93	0.48–1.62	45	0.79	0.57–1.05	31	0.62	0.42–0.89	5	0.69	0.22–1.61
Testis	1	0.71	0.00–4.08	4	0.81	0.21–2.08	3	1.15	0.22–3.40	0		
Kidney	11	5.71	2.83–10.25	8	1.02	0.43–2.01	4	0.65	0.17–1.67	1	1.09	0.00–6.24
Urinary bladder	3	0.92	0.17–2.71	19	1.42	0.85–2.22	12	1.10	0.57–1.93	1	0.63	0.00–3.62
Melanoma	3	0.66	0.12–1.96	7	0.38	0.15–0.80	6	0.45	0.16–0.99	3	1.65	0.31–4.89
Skin, squamous cell	6	2.50	0.90–5.49	18	1.88	1.11–2.97	14	1.79	0.97–3.01	5	4.62	1.46–10.88
Nervous system	7	2.24	0.89–4.65	15	1.23	0.69–2.03	8	0.91	0.39–1.79	0		
Thyroid gland	1	1.17	0.00–6.70	2	0.60	0.06–2.19	1	0.43	0.00–2.47	2	6.44	0.61–23.67
Endocrine glands	4	2.91	0.76–7.52	8	1.47	0.63–2.91	1	0.25	0.00–1.41	0		
Connective tissue	1	1.76	0.00–10.06	5	2.26	0.71–5.31	1	0.63	0.00–3.64	0		
Non‐Hodgkin's lymphoma	12	4.38	2.25–7.67	25	2.27	1.47–3.36	14	1.66	0.90–2.79	0		
Myeloma	4	4.71	1.23–12.19	5	1.44	0.45–3.38	3	1.08	0.20–3.18	1	2.48	0.00–14.24
Leukemia	6	2.66	0.96–5.84	9	0.99	0.45–1.88	4	0.57	0.15–1.47	0		
All cancer	252	3.33	2.93–3.77	520	1.67	1.53–1.82	362	1.46	1.32–1.62	52	1.46	1.09–1.92

Bold type indicates that the 95% CI does not include 1.00.

Pyrs, person‐years; O, observed; SIR, standardized incidence ratio; CI, confidence interval.

Person‐years for <1, 1–4, 5–9, 10+ are 24153, 89187, 56666, and 7203.

## Discussion

To the best of our knowledge, this population‐based cohort study on 29,271 patients diagnosed with HCV infection is a first nationwide attempt to systematically explore the subsequent risk for 35 common cancer sites/types using unified data from several nationwide Swedish registers. Increased risks were shown for six cancers, with SIRs ranging from 2.06 to 36.67, and the highest risk was for liver cancer. However, when stratified by duration of HCV infection, 5 of 6 cancer sites lost significance due to the limited number of patients, leading to wide confidence intervals.

Several studies reported the increased risk for liver cancer and NHL after HCV infection 14, 15, 16. The relative risk for developing liver cancer in patients with serologically confirmed HCV infection is 17, compared to 2.5 for HCV‐associated NHL 17. The individuals with HCV infection have a 1–4% annual risk to develop HCC 18. The risk was highest during the first year of follow‐up, suggesting the potential role of surveillance bias 19, as most of these cancer patients could do blood tests with the possibility of detecting HCV infection.

Worldwide, HCC is the third leading cause of cancer‐related mortality. Chronic HCV infection leads to the increased incidence of HCC in Western countries and Japan 5. The progression to HCC is likely to be determined by a combination of virus‐specific, host genetic, environmental, and immune‐related factors. The pathogenesis of HCC is a combination of direct and indirect mechanisms, which results from chronic oxidative damage that promotes the development of mutations. HCV encoded core, nonstructural protein 5A (NS5A), and NS3 directly promote HCC by altering host gene expression, while immune‐mediated inflammation contributes indirectly to tumorigenesis 20. Sustained proliferative signaling indirectly promotes virus replication through successive bouts of hepatitis, resulting in liver damage without virus clearance. HCV encodes core, NS3 and NS5A that promote liver cell proliferation via the *β*‐catenin pathway. HCV core promotes cyclin E and cdk2 expression. Both core and NS3 also activate multiple signal transduction pathways that promote cell growth 20. The prevalence of HCV in men is almost twice of the women 21, which was in line with our study population. Men usually have higher rates of lifetime drug‐related risk behaviors, such as needle use and sharing, whereas women have higher rates of lifetime sexual risk behaviors 21. However, the risk of subsequent cancer was largely consistent irrespective of gender, suggesting that the observed association between HCV and cancers might be not due to the risk factors associated with HCV, but due to HCV infection itself.

The risks for the cancers of the upper aerodigestive tract, esophagus, and lung were elevated. Tobacco exposure and alcohol use may explain somewhat of the excess risk above 22. It is known that individuals with drug injection have a very high risk of HCV infection, and these individuals are usually associated with excess alcohol drinking and cigarette smoking, which are the established causal factors for cancers in the lung, larynx, oral, larynx, and esophagus 22.

Jan P‐O mentioned that the HCV‐induced lymphomagenesis could be understood by using three general theories: (1) consecutive external stimulation of lymphocyte receptors by viral antigens and continuous proliferation; (2) HCV replication in B cells with oncogenic effect mediated by intracellular viral proteins; (3) permanent B‐cell damage (e.g., mutation of tumor suppressor genes) caused by a transiently intracellular virus (“hit and run” theory) 8.

The strengths of this study include the long follow‐up time, almost full coverage of the Swedish patients with HCV infection, and all the results obtained from a single population of individuals with HCV infection in a country, which can provide high medical standards and affordable health care. Based on the compulsory clinical reports by pathologists or cytologists (guaranteeing diagnostic accuracy and complete coverage at a national level), the identification of incident cases of cancer and detailed tumor information was provided by the Swedish Cancer Registry 23. As hospitalized patients are seen by many doctors, diagnoses of HCV infection are of high quality. The overall diagnostic accuracy in the Swedish Hospital Discharge Register is 88–90% on main diagnoses 24.

There are several limitations in this study. First, cases of HCV infection from primary health care only recorded from two regions of Sweden (Stockholm and Skåne County). Second, the information on important risk factors for cancer (e.g., diet and smoking) and detailed medical treatment for HCV infection is absent in our database, which may result in residual confounding. However, the socioeconomic status was adjusted for in this study, which may partly decrease the confounding due to smoking and physical inactivity. Moreover, the chance in the process of multiple comparisons may lead to some findings in this study.

In conclusion, the increased risks were shown for six cancers after HCV infection. The highest risk was for liver cancer. Further studies are needed to explore the endogenous and exogenous factors that lead to these associations.

## Conflict of Interest

The authors declare no conflict of interest.

## References

[1] Perz, J. F. , G. L. Armstrong , L. A. Farrington , Y. J. Hutin , and B. P. Bell . 2006 The contributions of hepatitis B virus and hepatitis C virus infections to cirrhosis and primary liver cancer worldwide. J. Hepatol. 45:529–538. 16879891 10.1016/j.jhep.2006.05.013

[2] Hatzakis, A. , S. Wait , J. Bruix , M. Buti , M. Carballo , M. Cavaleri , et al. 2011 The state of hepatitis B and C in Europe: report from the hepatitis B and C summit conference*. J. Viral Hepatitis 18(Suppl 1):1–16. 10.1111/j.1365-2893.2011.01499.x 21824223

[3] Mangia, A. , and L. Mottola . 2012 What's new in HCV genotype 2 treatment. Liver Int. 32(Suppl 1):135–140. 22212584 10.1111/j.1478-3231.2011.02710.x

[4] Smittskyddsinstitutet . 2010 Epidemiologisk årsrapport, 2009 .Avaliable at: https://www.folkhalsomyndigheten.se/pagefiles/14998/epidemiologisk-arsrapport-2009.pdf, 2010.

[5] Kim, M. N. , B. K. Kim , and K. H. Han . 2013 Hepatocellular carcinoma in patients with chronic hepatitis C virus infection in the Asia‐Pacific region. J. Gastroenterol. 48:681–688. 23463401 10.1007/s00535-013-0770-9 PMC3698419

[6] Kuper, H. A. H. , and D. Trichopoulos . 2000 Infections as a major preventable cause of human cancer. J. Intern. Med. 248:13. 10971784 10.1046/j.1365-2796.2000.00742.x

[7] Fiorino, S. , A. Cuppini , G. Castellani , M. L. Bacchi‐Reggiani , and E. Jovine . 2013 HBV‐ and HCV‐related infections and risk of pancreatic cancer. JOP 14:603–609. 24216545 10.6092/1590-8577/1948

[8] Peveling‐Oberhag, J. , L. Arcaini , M. L. Hansmann , and S. Zeuzem . 2013 Hepatitis C‐associated B‐cell non‐Hodgkin lymphomas. Epidemiology, molecular signature and clinical management. J. Hepatol. 59:169–177. 23542089 10.1016/j.jhep.2013.03.018

[9] Yang, J. D. , and L. R. Roberts . 2010 Hepatocellular carcinoma: a global view. Nat. Rev. Gastroenterol. Hepatol. 7:448–458. 20628345 10.1038/nrgastro.2010.100 PMC3926946

[10] Parkin, D. M. , F. Bray , J. Ferlay , and P. Pisani . 2005 Global cancer statistics, 2002. CA Cancer J. Clin. 55:74–108. 15761078 10.3322/canjclin.55.2.74

[11] El‐Serag, H. B. , and K. L. Rudolph . 2007 Hepatocellular carcinoma: epidemiology and molecular carcinogenesis. Gastroenterology 132:2557–2576. 17570226 10.1053/j.gastro.2007.04.061

[12] Sundquist, K. , J. Sundquist , and J. Ji . 2014 Risk of hepatocellular carcinoma and cancers at other sites among patients diagnosed with chronic hepatitis B virus infection in Sweden. J. Med. Virol. 86:18–22. 24038002 10.1002/jmv.23754

[13] Duberg, A. , R. Janzon , E. Back , K. Ekdahl , and A. Blaxhult . 2008 The epidemiology of hepatitis C virus infection in Sweden. Euro. Surveill. 13:18882. 18761966 10.2807/ese.13.21.18882-en

[14] Shi, J. , L. Zhu , S. Liu , and W. F. Xie . 2005 A meta‐analysis of case‐control studies on the combined effect of hepatitis B and C virus infections in causing hepatocellular carcinoma in China. Br. J. Cancer 92:607–612. 15685242 10.1038/sj.bjc.6602333 PMC2362087

[15] Matsuo, K. , A. Kusano , A. Sugumar , S. Nakamura , K. Tajima , and N. E. Mueller . 2004 Effect of hepatitis C virus infection on the risk of non‐Hodgkin's lymphoma: a meta‐analysis of epidemiological studies. Cancer Sci. 95:745–752. 15471561 10.1111/j.1349-7006.2004.tb03256.x PMC11159764

[16] Dal Maso, L. , and S. Franceschi . 2006 Hepatitis C virus and risk of lymphoma and other lymphoid neoplasms: a meta‐analysis of epidemiologic studies. Cancer Epidemiol. Biomark. Prev. 15: 2078–2085. 10.1158/1055-9965.EPI-06-0308 17119031

[17] de Martel, C. , J. Ferlay , S. Franceschi , J. Vignat , F. Bray , D. Forman , et al. 2012 Global burden of cancers attributable to infections in 2008: a review and synthetic analysis. Lancet Oncol. 13:607–615. 22575588 10.1016/S1470-2045(12)70137-7

[18] Ghany, M. G. , D. B. Strader , D. L. Thomas , and L. B. Seeff . 2009 American Association for the Study of Liver D. Diagnosis, management, and treatment of hepatitis C: an update. Hepatology 49:1335–1374. 19330875 10.1002/hep.22759 PMC7477893

[19] Ji, J. , X. Shu , X. Li , K. Sundquist , J. Sundquist , and K. Hemminki . 2009 Cancer risk in hospitalized sarcoidosis patients: a follow‐up study in Sweden. Ann. Oncol. 20:1121–1126. 19211624 10.1093/annonc/mdn767

[20] Mesri, E. A. , M. A. Feitelson , and K. Munger . 2014 Human viral oncogenesis: a cancer hallmarks analysis. Cell Host Microbe 15:266–282. 24629334 10.1016/j.chom.2014.02.011 PMC3992243

[21] Butterfield, M. I. , H. B. Bosworth , K. G. Meador , K. M. Stechuchak , S. M. Essock , F. C. Osher , et al. 2003 Gender differences in hepatitis C infection and risks among persons with severe mental illness. Psychiatric Serv.(Washington, DC) 54: 848–853. 10.1176/appi.ps.54.6.848 12773599

[22] Swart, A. , L. Burns , L. Mao , A. E. Grulich , J. Amin , D. L. O'Connell , et al. 2012 The importance of blood‐borne viruses in elevated cancer risk among opioid‐dependent people: a population‐based cohort study. BMJ Open 2:e00175. 10.1136/bmjopen-2012-001755 PMC3488729 23045358

[23] Ji, J. , K. Sundquist , J. Sundquist , and K. Hemminki . 2012 Comparability of cancer identification among Death Registry, Cancer Registry and Hospital Discharge Registry. Int. J. Cancer 131:2085–2093. 22307919 10.1002/ijc.27462

[24] Ludvigsson, J. F. , E. Andersson , A. Ekbom , M. Feychting , J. L. Kim , C. Reuterwall , et al. 2011 External review and validation of the Swedish national inpatient register. BMC Public Health 11:450. 21658213 10.1186/1471-2458-11-450 PMC3142234

